# ENVISION: A DIGITAL TOOL TO IMPROVE SYMPTOM MANAGEMENT AMONG OLDER ADULTS WITH ADVANCED CANCER

**DOI:** 10.1093/geroni/igad104.1755

**Published:** 2023-12-21

**Authors:** Karla Washington, Allison Donehower, Debra Parker Oliver, Jacquelyn Benson, George Demiris

**Affiliations:** Washington University in St. Louis, St. Louis, Missouri, United States; University Of Missouri, Columbia, Missouri, United States; Washington University in St. Louis, St. Louis, Missouri, United States; Washington University in St. Louis, St. Louis, Missouri, United States; University of Pennsylvania, Philadelphia, Pennsylvania, United States

## Abstract

Hospice is a largely community-based healthcare service provided by an interdisciplinary team of professionals who partner with patients and their family members to control pain and other distressing symptoms commonly encountered at end of life. Research has shown that, despite receipt of hospice care, older patients with advanced cancer experience high rates of symptom distress. This presentation will describe the human-centered design of ENVISION (ENgagement and Visualization to Improve Symptoms In ONcology care), a digital health solution designed to improve hospice symptom management by strengthening patients’ and families’ symptom management self-efficacy and increasing the caregiver-centeredness of communication with hospice teams. Particular emphasis will be placed on design considerations adopted to enhance the digital inclusivity of the tool, resulting from research conducted to address the following research question: To what extent is ENVISION accessible, adoptable, and applicable to the strengths and needs of older hospice patients and their family caregivers? In answering this question, researchers conducted a multi-method qualitative study during which 34 users (including hospice nurses, social workers, physicians, chaplains, and family caregivers) were individually observed using the tool and interviewed about their experiences. Data were analyzed via conventional content analysis. Findings informed recommendations to address both digital and health literacy concerns, simplify data visualizations, and focus more specifically on high-priority symptoms and other wellbeing indicators, resulting in a more digitally inclusive tool with promise to improve the lives of older adults with advanced cancer and the family members who care for them.

